# Passing path predicts shooting outcome in football

**DOI:** 10.1038/s41598-024-60183-7

**Published:** 2024-04-26

**Authors:** Shun Cao

**Affiliations:** Department of Information Science Technology, University of Houston, Houston, TX 77204 USA

**Keywords:** Ball movement, Passing path, Performance, Shooting outcome, Soccer, Computational science, Information theory and computation

## Abstract

What determines the outcome of a shot (scored or unscored) in football (soccer)? Numerous studies have investigated various aspects of this question, including the skills and physical/mental state of the shooter or goalkeeper, the positional information of shots, as well as the attacking styles and defensive formations of the opposing team. However, a critical question has received limited attention: How does the passing path affect the outcome of a shot? In other words, does the path of the ball before shooting significantly influence the result when the same player takes two shots from the same location? This study aims to fill the gap in the literature by conducting qualitative studies using a dataset comprising 34,938 shots, along with corresponding passing paths from top-tier football leagues and international competitions such as the World Cup. Eighteen path features were extracted and applied to three different machine-learning models. The results indicate that the passing path, whether with or without the positional information of shots, can indeed predict shooting outcomes and reveal influential path features. Moreover, it suggests that taking quick actions to move the ball across areas with a high probability of scoring a goal can significantly increases the chance of a successful shot. Interestingly, certain path features that are commonly considered important for team performance, such as the distribution of passes among players and the overall path length, were found to be less significant for shooting outcomes. These findings enhance our understanding of the effective ball-passing and provide valuable insights into the critical factors for achieving successful shots in football games.

## Introduction

Football, or soccer, is a team sport characterized by its dynamics and complexity, where players constantly interact with each other by either cooperating with team members or competing against opponent players within a system^1,2^. The performance of a football team depends heavily on the ability to synchronize collective movements, execute accurate passes, create scoring opportunities, and organize an effective defense^3,4^. It is reasonable to assert that a football team’s performance emerges from the complex and fundamental interactions among all players from both sides^5,6^. In recent years, the study of the performance of football teams has increasingly embraced advanced analytical techniques and data-driven approaches using positional data, event logs, and statistical information about teams and players^7–9^. Researchers and sports analysts have recognized the significant factors that influence team behaviors and performance through various studies such as passing networks^10^, dynamic analysis^11,12^, discovery of tactical strategies^13^, etc.

To significantly increase the likelihood of winning football games, it is crucial to grasp the mechanics of successful shots and enhance shooting outcome. This particular concern has led to the emergence of a widely discussed research area known as the “expected goals model”^14,15^. This model aims to estimate the probability of any given shot converting into a goal, considering various factors related to the shot itself, such as the shooting position, angle, and shooting techniques, among others. Furthermore, numerous previous research endeavors have delved into various facets of shooting outcomes, including the techniques employed by shooters, the skill levels of goalkeepers, and the psychological and physical factors impacting players’ performances during matches^16–18^. Furthermore, some studies also revealed that the formation of both defending and attacking teams significantly influences the outcomes of shooting attempts. These studies leverage positional information about players at the moment of the shot^19^. However, it’s imperative to acknowledge that the effectiveness of a shot is not solely determined by these factors. Collective movements of players from both sides leading up to the shooting action also play a substantial role^3,20^. In some measure, the final shooting position, the defensive formation, and the states of the shooter, goalkeeper, and other players at the moment of the shot are all closely linked to preceding ball passing. The dynamics of ball passing, including actions such as kicking, passing, dribbling, and more, are orchestrated by attacking players and influenced by the overarching strategies of both attacking and defending teams^21,22^. This involves dynamic shifts in players’ positions, the attention of defenders, and a myriad of detailed movement and decision-making processes. Consequently, comprehending the impact of passing paths or the trajectories of ball movement before shooting actions may unveil critical passing patterns that shed light on the reasons behind successful shots. Such insights can significantly enhance a football team’s overall performance.

To investigate the passing paths in football games, numerous studies adopted the method of passing network modeling and analysis, which analyze the complex structure regarding who passes the ball to whom or how the ball moves from one zone to another. These studies have identified various topological patterns, strong and weak connections among players, positional relationships, and insights into tactics and player/team performance^23,24^. However, surprisingly little attention has been given to examining the impact of passing networks on the outcomes of individual shots instead of the overall team performance. This gap in knowledge may be attributed to the infrequent occurrence of passing sequences or the scarcity of positional information in each possession by a team. As a result, when employing the passing network method, there is a scarcity of topological information available for in-depth analysis. Though a handful of research has shed light on the effects of passing path or ball movement on the shooting outcome based on various approaches^19,25,26^, none of them have addressed the fundamental question of which types of passing paths are linked to successful shots, i.e., scoring goals.

This paper aims to systematically address this gap in football literature. The primary objective is to build a connection between the passing path and shooting outcomes and then to identify the critical features of these paths that significantly influence the shooting outcome. To tackle this challenge, this work uses a wide range of features extracted from the passing paths. Three machine learning models, namely Logistic Regression, Random Forest, and Artificial Neural Network were employed to demonstrate how these extracted path features can be effectively utilized for modeling and predicting shooting outcomes. Furthermore, by estimating the importance of path features, this work offers valuable guidance to coaches and sports analysts, empowering them to identify and design effective passing paths and improve the performance of football teams.

## Empirical data

This study utilized real-world event logs from top-tier football leagues and international competitions, including the World Cup. The event logs were obtained from Wyscout^7^, and further details can be found at https://apidocs.wyscout.com/. The data sets comprise a wide range of sequential events that occur during football matches. They provide valuable information such as event types (e.g., pass, foul, duel, shot), subevent types (e.g., simple pass, smart pass, head pass, hand pass, launch), additional event details (e.g., accuracy), event time (measured in seconds since the start of the current half), player ID (identifying the event generator), position (origin and destination coordinates of the event), and more. Table 1 gives two examples from the data sets to illustrate its major structure.

**Table 1:** Examples of the event data^7^.

Example one	Example two
“eventID”: 8,	“eventID”: 1,
“eventName”: “Pass”,	“eventName”: “Duel”,
“eventSec”: 3.889375,	“eventSec”: 15.685687,
“id”: 100297,	“id”: 263885674,
“matchId”: 2058017,	“matchId”: 2058017,
“matchPeriod”: “1H”,	“matchPeriod”: “1H”,
“playerId”: 69968,	“playerId”: 3309,
“positions”: [{“y”: 52, “x”: 39}, {“y”: 74, “x”: 34}],	“positions”: [{“y”: 49, “x”: 36}, {“y”: 80, “x”: 37}],
“subEventId”: 85,	“subEventId”: 13,
“subEventName”: “Simple pass”,	“subEventName”: “Ground loose ball duel”,
“tags”: [{“id”: 1801}]	“tags”: [{“id”: 701}, {“id”: 1802}]
“teamId”: 9598	“teamId”: 4418

**Figure 1:**
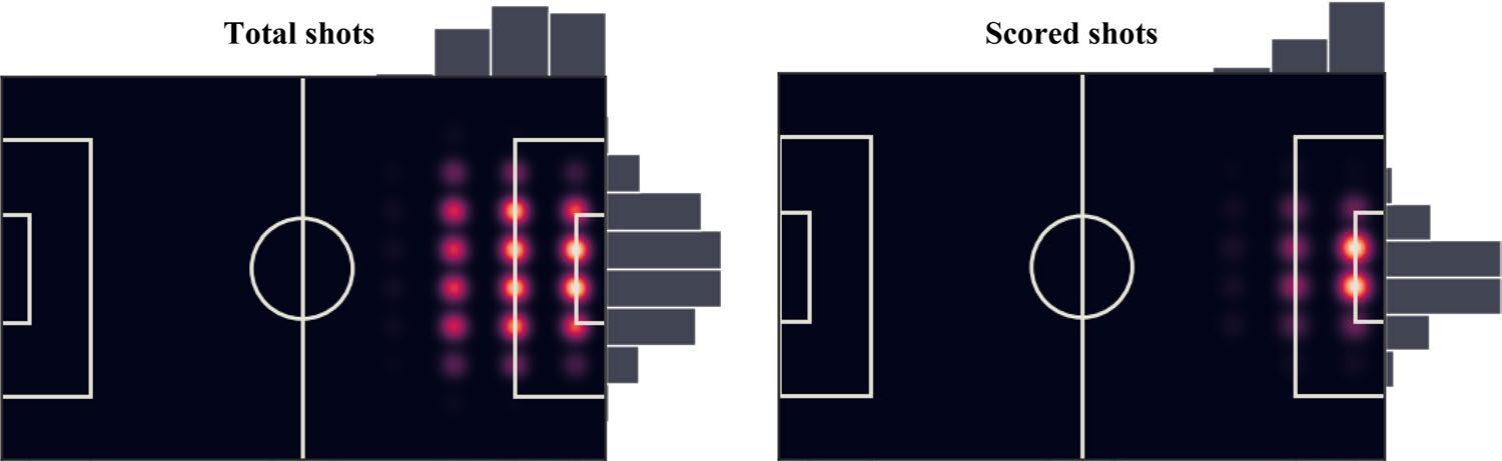
Shot position distribution on the football field.

In total, this study analyzed 1,941 football matches, which included 64 matches from the 2018 World Cup, 51 matches from the 2016 UEFA European Football Championship, 306 matches from the 2017–2018 Germany Bundesliga, 380 matches from the 2017–2018 England Premier League, 380 matches from the 2017–2018 France Ligue 1, 380 matches from the 2017–2018 Italy Serie A, and 380 matches from the 2017–2018 Spain La Liga. The overall data set regarding shots consists of 34,938 data points (the shooting outcomes and the corresponding passing paths), including 3,931 scored shots and 31,007 unscored shots. Those paths ending with shots that involved only one event (e.g., penalties, free kick shots) were not considered in this study. Figure 1 provides the shot position distribution on the football field.

## Passing path and shooting outcome

**Figure 2:**
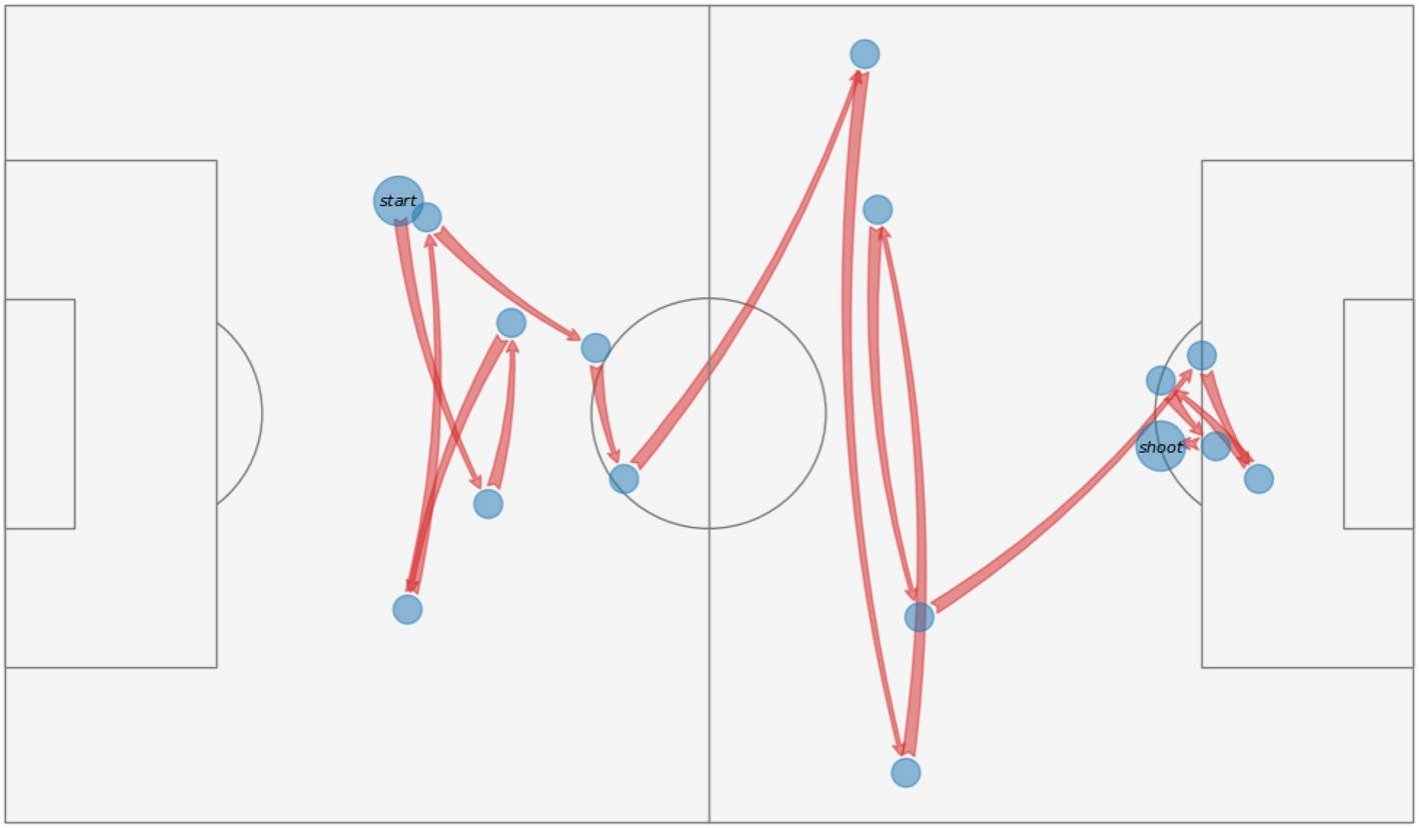
A continuous passing path during team possession.

In this study, the term “passing path” refers to the trajectory or route taken by the ball during a team’s continuous possession of the ball on the football field, encompassing actions such as passes, free kicks, dribbles, duels, and more. It is important to note that the ball movement during a possession is not a continuous trajectory, as positional information is extracted from non-continuous event logs. Additionally, events, where the opposing team touches the ball or engages in a duel that doesn’t result in a change of ball control, are still considered part of the team's continuous possession. The passing path incorporates various spatial and temporal information, influenced by factors such as the individual abilities of the players, team cooperation, the attacking tactics performed by the football team, and the overall game situation. This path can also significantly impact the collective movement of defenders (i.e., the opposing team), their defending formation, attention, and decision-making processes. To illustrate, Fig. 2 presents an example of a passing path in a football match, with nodes of “start” and “shoot” representing the starting position of the team’s possession and the final shooting area. The shooting outcome refers to the result of a player’s attempt to score a goal by shooting the ball toward the opponent’s net. It can be classified as either scored, meaning the ball successfully enters the net, or unscored, indicating that the shot misses the target or is blocked by the goalkeeper or other defenders. Given the absence of standard football pitch dimensions, a unified approach considers all pitches as 105 m in length and 68 m in width according to FIFA football stadiums guidelines (https://publications.fifa.com/en/football-stadiums-guidelines/technical-guideline/stadium-guidelines/pitch-dimensions-and-surrounding-areas/).

### Extracting passing path features

The primary objective of this study is to utilize machine learning models to examine how passing paths impact shooting outcomes. This requires extracting and quantifying crucial and relevant information about the passing paths. The extracted features are also required to be applicable to the prediction models. To achieve this, some established metrics that measure passing paths or passing networks were incorporated into this work, which have been proven to have a strong relationship with team performance. Furthermore, several features, such as *attack intensity* and *y range*, are first proposed in this work. Some general measures of passing paths, such as path length and speed, were also considered. In total, eighteen features were extracted from the passing path, each of which is described in detail below.

#### Overall path length

The *overall path length* refers to the distance traveled by a ball from the starting position to the shooting position during a team’s possession. It has been observed that the overall path length is positively correlated with the shooting outcome, suggesting that goals are more likely to be scored from longer overall paths compared to shorter ones^27^. To calculate this length, the dimensions of the football field are necessary. Since there is no standardized dimension for qualified football fields in the available data sets, this study adopts the dimensions of a widely recognized football field, measuring 105 m in length and 68 m in width. The length of the passing path is computed using the Euclidean distance formula.

#### Ratio of passing length

The *ratio of passing length* measures the proportion of passing distance within the overall path length. In addition to passing actions, ball movement can also be brought by actions like dribbling or dueling. This ratio acts as a means of evaluating the extent to which the passing movement contributes to shooting outcomes, in comparison to other types of ball movement.

#### Shot distance

*Shot distance* is defined as the distance from the shooting position to the center of the target. It serves as a precise measure of the shooter’s proximity to the intended target, holding significant importance in determining the outcome of the shot. This fundamental feature has been extensively studied and proven to play a crucial role in shooting accuracy and effectiveness^25,28^.

#### Start distance

Similar to the shot distance, the start distance is a measure of the initial distance between the ball’s starting point on its trajectory and the center of the attacking target.

#### Average distance

The average distance is determined by calculating the mean distance between all recorded location points of a passing path and the center of the attacking target.

#### X range

A passing path involves a series of positions on the football field, denoted as $\left({x}_{i}, {y}_{i}\right) i\le n$, where $i$ represents the position index and $n$ is the total number of positions. The *x range* of a passing path is defined as the difference between the maximum and minimum x coordinates in the path, which can be expressed as:

1 $$x \; range={\text{max}}\left({x}_{i}\right)-{\text{min}}({x}_{i})$$

#### Y range

The Y range is defined in the same manner as the *x range*, with the following formula:

2 $$y \; range={\text{max}}\left({y}_{i}\right)-{\text{min}}({y}_{i})$$

#### Moving directness

*Moving directness* quantifies the degree of directness of the ball’s movement from its starting position to the final shot position. It is calculated by dividing the straight-line distance between the starting point and the shot point by the *overall path length*. A higher moving directness value indicates a more direct movement of the ball towards the shot position.

#### Possession time

*Possession time* refers to the duration that a team maintains control of the ball during a match or a specific possession. It serves as a crucial metric for evaluating a team’s performance and style of play^29,30^. In this work, possession time indicates the duration of a passing path. Research has revealed that successful teams tend to have significantly longer possessions compared to unsuccessful teams, regardless of match outcomes^31^.

#### Overall moving speed

The *overall moving speed* refers to the average speed at which the ball moves along its path from the starting location to the shooting position. This characteristic, especially for the attacking and counter-attacking periods, has been identified as strongly correlated with a football team’s attacking style and performance^32^.

#### Direct speed

*Direct speed* is a metric that quantifies the velocity of a ball’s movement along a straight line from its starting point to the shooting point, which can be seen as an extending index of the *moving directness*. It provides an indication of how quickly the ball travels from the starting point to the shooting point. The direct speed is calculated as ${L}_{s}/T$, where ${L}_{e}$ represents the straight-line distance between the starting point and shooting point of the ball's trajectory, and $T$ denotes the total *possession time*.

#### Passing ratio

*Passing ratio*, also known as network intensity, is a crucial metric extensively employed in studies on passing networks in football games. It is defined as the total number of passes among all players in a team divided by the duration of time the team possesses the ball during a given period. This metric has been found to have a positive correlation with team performance^15,23^. Here, the *passing ratio* was adopted to measure the efficiency of a passing path by calculating the number of passes relative to the time of possession of this passing path.

#### Shot index

**Figure 3:**
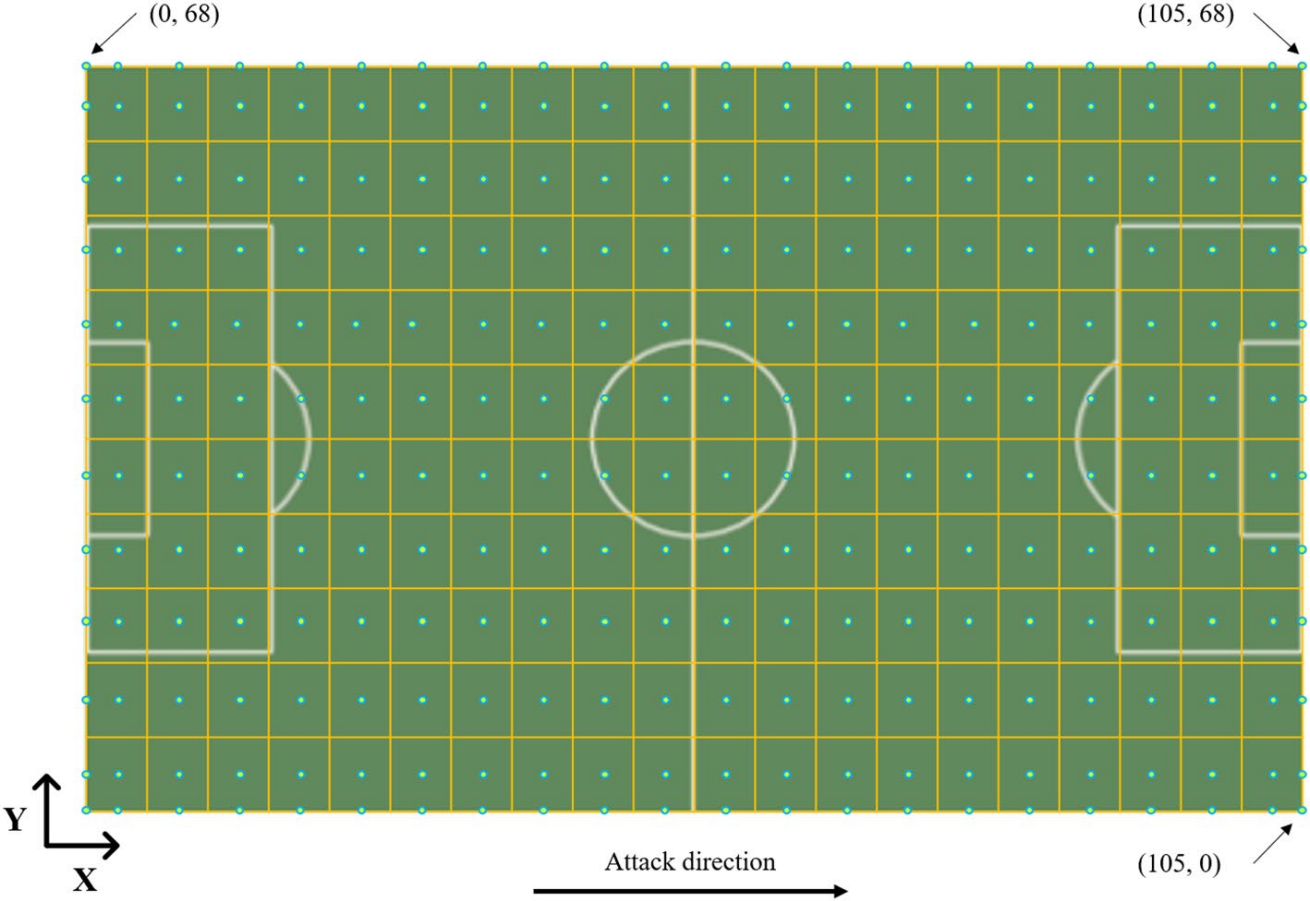
Division of the football field.

The *shot index* of a specific location on the football field is a quantifiable measure representing the likelihood of scoring a goal from that position. This metric provides a more objective and insightful assessment of a position’s significance in football games, as it indicates the probability of a successful goal attempt. A position with a high *shot index* may be the one where attacking players are more inclined to distribute the ball, and defensive players are likely to allocate extra defending attention. This study used a method based on the approach in^33^, which includes dividing the football field into zones and statistically evaluating the goal-scoring opportunities. However, the field division Rathke’s work is somewhat coarse-grained, as the attacking half of the football field (approximately 1,884 square meters) was divided into only eight zones. This coarse division may introduce bias in the analysis. Therefore, considering the substantial amount of available shot information in this work (34,938 shots in total), it is possible to investigate a more detailed shot index. To achieve this, the entire football field was divided into 200 areas, comprising 10 corridors (vertical direction) and 20 sectors (horizontal direction), as illustrated in Fig. 3. Each shot is assigned to a specific zone, and the number of goals, and the total shots in each area are computed as percentages, serving as the shot index for that particular area. Formally, the *shot index* (${sIndex}_{i}$) of area $i$ in the football field is defined as:

3 $${sIndex}_{i}=\frac{{G}_{i}}{TS}$$

where ${G}_{i}$ and $TS$ represent the number of goals (scored shots) and total shots, respectively. Furthermore, for shots occurring within an area, this work considers the center of that area as the location point, marked as dots. Additionally, the four boundaries of the football field were also divided into distinct sections, aligned with the division of the football field, as depicted in Fig. 3.

#### Acceleration index

This metric serves as an indicator of a team’s speed to obtain the chance of shooting. Originally introduced in a study conducted by Pappalardo et al.^7^, it is calculated by dividing the highest *shot index* within the involved areas by the square of the *possession time*. This index has been found to have a strong correlation with team performance^7^. In this context, the *shot index* of the shooting area is employed instead of the highest shot index along the passing path.

#### Attack intensity

*Attack intensity* is a critical metric to define a passing path in football games. It is determined by how quick actions the attacking team takes and where these actions occur on the football field. The calculation of this metric involves considering the positions of all events (e.g., passes, duels, shots, touches) within the ball’s movement path. *Attack intensity* ($AI$) of a passing path is computed as the sum of the shot indexes of all the involved positions ($\sum sInde{x}_{k}$) divided by the overall possession time ($T$) in a passing path:

4 $$AI=\frac{\sum sInde{x}_{k}}{T}$$

This means that when an attacking team executes rapid actions such as passing and dribbling to advance the ball through areas with a high likelihood of scoring a goal, the overall *attack intensity* of their movement along this path, during a single possession, is considered high. Additionally, passing paths exhibiting a higher *attack intensit*y are more likely to generate scoring opportunities. There are two reasons for this. Firstly, these paths often involve positions where successful shots have a higher probability. Secondly, they present challenges for defenders who must defend against multiple positions within a shorter timeframe while remaining attentive. However, it is important to note that this assumption requires validation through further analysis, which was provided in “Results and discussion” section of this paper.

#### Number of players

The number of players involved in the passing path has been shown to have a substantial influence on the outcome of shooting^23,34^. It is worth noting that even rare instances in which players from the opposing team interact with the ball (e.g., touch) during a possession are also taken into consideration in this feature.

#### Centralization of passing actions

*Centralization of passing actions* measures the level of inequality in the distribution of passing actions among different players. A handful of studies regarding passing networks in football concluded that a team’s overall centralization of the passing network (weighted by the number of passes) is highly linked to team performance^23,35,36^. Though the passing path does not incorporate the complex passing structure as those studies, the distribution passing actions might also link to the shooting outcome. Therefore, the centralization of passing actions was also incorporated in this study, which is defined as:

5 $${C}_{n}=\frac{{\sum }_{i=1}^{n}(N({p}^{*})-N({p}_{i}))}{N({p}^{*})\times (n-1)}$$

where ${C}_{n}$ indicates the general *centralization of passing actions* of a passing path. $N({p}^{*})$ is the maximum number of passing actions conducted by one player, ${p}^{*}$, comparing with others, who involved in the passing path, while $N({p}_{i}))$ represents the number of passes conducted by player ${p}_{i}$.

#### Centralization of possession time

The concept of *centralization of possession time* pertains to quantifying the degree of inequality in the distribution of possession time among various players in the period of a passing path. It shares similarities with the use of possession time as edge weight in passing networks to determine network centralization^23,35,36^. Here, it is defined as follows.

6 $${C}_{t}=\frac{{\sum }_{i=1}^{n}(T({p}^{*})-T({p}_{i}))}{T({p}^{*})\times (n-1)}$$

where ${C}_{t}$ indicates the general centralization of possession of a passing path. $T({p}^{*})$ is the maximum ball-possession time performed by one player, ${p}^{*}$, comparing with others, who involved in the passing path, while $T({p}_{i}))$ represents the ball-possession time performed of player ${p}_{i}$.

This study presents a pioneering analysis of the effects of passing paths on shooting outcomes, introducing a range of innovative features aimed at capturing their dynamic nature. For example, *attack intensity* and *direct speed* are two of the novel features. Others, such as *start distance*, *centralization of possession time*, and *average distance*, are expanded from previously reported studies. While some reported features have been touched upon in prior research and shown to impact team performance or play styles, the passing path itself remains a reservoir of hidden information. This is particularly concerning the dynamic and intricate interactions among the ball and players at different times and locations, which can significantly influence shooting outcomes.

Table 2 gives the descriptive statistics for all extracted features from the passing path, including the mean, standard deviation (S.D.), minimum (Min), and maximum (Max) values. Among these features, some represent real-world physical values such as overall path length, start distance, shot distance, possession time, and overall moving speed. Other ratios, such as moving directness and shot index, are mainly constrained to values between 0 and 1. It is important to note that the maximum number of players is 14, as this calculation also included players from the defending team who touched the ball. Notably, the maximum overall moving speed recorded is 499.9 m/s, which appears unrealistic in football games. This discrepancy may be attributed to a few instances of inaccurate data recording, such as an erroneous time label for a quick pass. However, these data points were still included in the analysis as they did not affect the final conclusions of the study.

**Table 2:** Overview of the features used in this study.

Features	Description	Mean	S.D	Min	Max
*Overall path length (m)*	Ball travel distance from starting position to shooting position during a team’s possession	364.2	344.4	2.100	4168
*Ratio of passing length*	Proportion of passing distance within the overall path length	0.4248	0.2105	0.000	1.000
*Start distance (m)*	Distance between starting position and the center of attacking target	54.59	22.84	1.251	111.3
*Shot distance (m)*	Distance from the shooting position to the center of attacking target	18.04	8.189	0.680	102.3
*Average distance (m)*	Mean distance between all recorded locations of a passing path and center of attacking target	37.03	13.77	2.625	98.96
*x range (m)*	Difference between the maximum and minimum *x* coordinates in the path	44.60	26.94	0.000	105.0
*y range (m)*	Difference between the maximum and minimum *y* coordinates in the path	48.49	14.81	0.000	68.00
*Moving directness*	Ratio of straight-line distance from starting position to shot position to overall path length	0.2154	0.2964	0.000	23.25
*Possession time (s)*	Duration of a passing path	15.48	13.71	0.07268	228.9
*Overall moving speed (m/s)*	Average speed of ball movement in a passing path	21.17	12.39	0.000	499.9
*Direct speed (m/s)*	Velocity of a ball’s movement along a straight line from its starting position to shooting position	4.661	4.092	0.000	102.6
*Passing ratio*	Total number of passes divided by possession time	0.3093	0.1494	0.000	2.931
*Number of players*	Number of players involved in one possession	4.906	2.267	1	14
*Centralization of possession time*	Degree of inequality of possession time among various players in the possession time	0.5584	0.2016	0.000	1.000
*Centralization of passing actions*	Level of inequality of passing actions among different players in a passing path	0.3896	0.2839	0.000	1.000
*Shot index*	Likelihood of scoring a goal from a given position	0.04847	0.04824	0.000	0.1475
*Acceleration index*	Ratio of shot index of shooting location to the square of the possession time	0.01751	0.02252	0.000	0.2535
*Attack intensity*	Sum of the *shot indexes* of all positions in a passing path divided by possession time	0.03101	0.07064	0.000	4.442

## Methods

In order to determine whether the path of ball movement can influence the outcome of shooting in football games, it is crucial to establish a connection between the shooting outcome (i.e., scored or unscored) and the aforementioned path features. Machine learning classification models offer a practical and valuable approach to assessing the impact of these features on the shooting outcome, enabling us to identify the key ball movement characteristics that contribute to shooting success. However, employing different models often yields distinct results. Consequently, relying on a single model poses the risk of underperformance or sensitivity to variations in the passing path data. Conversely, leveraging multiple machine learning models enables a more thorough exploration of the data, reduces the bias and variance of each individual model, increases interpretability, and bolsters the reliability and robustness of the predictive modeling process. Several reported studies have underscored the advantages of utilizing multiple models for predictive analysis in football data^23,37^. Therefore, this study employed three different models (either parametric or non-parametric), Logistic Regression^38^, Random Forest^39^, and Artificial Neural Network^40^, to test the hypothesis. The three models each offer unique advantages in data analysis. Logistic Regression provides a simple and interpretable model, ideal for understanding individual feature impacts and serving as a baseline. Random Forest excels in capturing complex relationships and interactions between features, making it robust against overfitting and suitable for handling both numerical and categorical data. Artificial Neural Networks offer unmatched flexibility in learning intricate patterns and high-dimensional relationships in the data, albeit at the expense of interpretability and increased computational complexity. Together, these models complement each other by combining the interpretability of Logistic Regression, the robustness of Random Forests, and the flexibility of Artificial Neural Networks to provide a comprehensive understanding of the data regarding the relationship between path features and shooting outcomes and improve predictive performance. The following section provides a description of the machine learning models utilized.

### Logistic Regression

This study employed the widely used conventional Logistic Regression, known as one of the most commonly applied generalized linear models. Its purpose is to establish a relationship between a group of independent variables, specifically the extracted features of passing paths, and a binary dependent variable, namely whether shots were scored or not. By utilizing this Logistic Regression model, this work aims to model the relationship between the path features and the shooting outcomes and further identifies the significant features influencing the shooting outcome in football games.

### Random Forest

Random Forest model is an ensemble learning and nonparametric method used for tasks such as classification, regression, and more. It operates by constructing a series of uncorrelated decision trees during training. In classification tasks, the Random Forest model outputs the class selected by the majority of the trees. One notable advantage of this machine learning approach over regression-based methods (e.g., Logistic Regression) is its independence from the order in which variables are entered into a stepwise model. Additionally, Random Forest model accounts for complex and nonlinear relationships between variables. Furthermore, it can directly assess the importance of each feature based on the relative contribution to predictions. This type of feature importance analysis provides valuable insights for the question of what types of passing paths are more effective and successful.

### Artificial Neural Network

Artificial Neural Networks (ANN) forms the foundation of deep learning algorithms. Drawing inspiration from the human brain, it emulates the intercommunication of biological neurons. These networks are renowned for their exceptional predictive capabilities in real-world scenarios. One key technique integral to training neural networks is backpropagation, which has significantly contributed to advancements in machine learning and artificial intelligence. In this study, the model of Backpropagation Feedforward Artificial Neural Network was also employed as the third prediction model.

The evaluation of these models focuses on their predictive capabilities in this work. Therefore, three widely recognized measures were adopted, accuracy, specificity, and sensitivity, to assess the effectiveness of the three classification models. These metrics provide valuable insights into the models’ ability to accurately predict the outcomes of the shooting.

Accuracy, a commonly used metric, quantifies the overall correctness of the model’s predictions. It is calculated by dividing the number of correct predictions by the total number of predictions, as defined below.

7 $$Accuracy=\frac{(tp+tn)}{(tp+tn+fp+fn)}$$

where $tp$, $tn$, $fp$, $fn$ refer to the number of positive instances correctly classified as positive, the number of negative instances correctly classified as negative, the number of negative instances incorrectly classified as positive (type I error), the number of positive instances incorrectly classified as negative (type II error), respectively.

Specificity, also known as the true negative rate, gauges a model’s proficiency in accurately recognizing negative instances. A higher specificity indicates a reduced likelihood of the model mistakenly categorizing negative instances. Its definition is as follows.

8 $$Specificity=\frac{tn}{tn+fp}$$

Sensitivity is also called Recall or True Positive Rate, which measures a model’s ability to correctly identify positive instances. A higher sensitivity indicates that the model has a lower likelihood of missing positive instances The formula for sensitivity is:

9 $$Sensitivity=\frac{tp}{tp+fn}$$

In addition to the aforementioned metrics, this study also utilized the AUC (Area Under the Curve) index to assess the predictive accuracy of the model^41^. The AUC index quantifies the balance between the model’s true positive rate (sensitivity) and its false positive rate. AUC is particularly valuable in binary classification scenarios as it offers a comprehensive evaluation of the model’s performance, summarizing its capacity to distinguish between positive and negative instances without relying on a specific decision threshold.

The data used in this study is unbalanced, with a significantly higher number of unscored shots (31,007) compared to scored shots (3931). To address this issue, the undersampling technique^42^ was employed, which involves reducing the number of instances from the majority class (unscored shots) to match the minority class (scored shots). In this study, all the data of scored shots were adopted and kept unchanged, while an equal number of unscored data were randomly sampled from the whole unscored dataset. To ensure minimal information loss, a Monte Carlo method was utilized, running the classification models multiple times (e.g., 50 times) using different sets of unscored data. Additionally, for each run, the cross-validation method^43^ was applied.

Moreover, identifying the key features that are more likely to result in successful shots is essential for understanding the effects of passing paths on shooting outcomes. This knowledge can be utilized by sports specialists, team coaches, or managers to devise effective attacking tactics. In the case of Random Forest model, each node in the decision tree represents a condition for splitting values in a particular feature. This split ensures that similar values of the dependent variable are grouped together. The condition is based on impurity, specifically Gini impurity^44^ in this work. The importance of a feature is determined by its contribution to reducing the weighted impurity. For the Artificial Neural Network, the study used the coefficients (i.e., weights) between inputs and outputs for each node in the hidden layers, which was described in the referenced study^45^. However, when it comes to logistic regression, there is no standardized approach for evaluating feature importance. As a rule of thumb, the magnitude of the coefficient and the p-value (which indicates the significance of a feature) can provide some coarse-grained references of their importance to a certain extent. Moreover, all the data processing and machine learning models were applied using the Python programming language version 3.9. The relevant Python code concerning data processing, generation of independent features, settings for each machine learning model, and data analysis is also accessible on GitHub (https://github.com/shun-cao?tab=repositories).

### Ethical clearance

This study employed data sourced from a publicly available dataset, accessible at, https://apidocs.wyscout.com/. Notably, the personally identifiable information of professional football players, coaches, referees are also public accessible. As such, ethical approval was not required for this study as it involved secondary analysis of anonymized data.

## Results and discussion

The Logistic Regression, Random Forest, and ANN models were implemented on a dataset extracted from passing paths and the shooting results, which have been specifically fine-tuned to ensure an equitable comparison of their results in this work. The prediction results in this section are all based on the average values obtained from fivefold cross-validation and 50 repetitions using different unscored datasets, totaling 250 runs for each model. A feature selection procedure was performed for the Logistic Regression model as it is sensitive to the issue of multicollinearity. The Recursive Feature Elimination (RFE) technique^46^, a popular tool for selecting features, was employed. RFE recursively considers smaller and smaller sets of features based on their importance. As a result, there are two sets of results for the Logistic Regression model: one obtained using all eighteen features, and the other obtained using the selected thirteen features based on RFE and Akaike Information Criterion (AIC)^47^. For consistency, all features were used in the Random Forest and ANN models, which are less likely to be suffered from multicollinearity problems.

### Initial prediction results

The initial prediction results obtained from different models are presented in Table 3, which considered the information regarding shooting positions. The accuracy and specificity of all the models are approximately 70%, while the sensitivity is either higher than or close to 70%. The AUC index for all the models is around 76%, indicating the reasonably good performance of the predictive models. Based on the prediction results in Table 3, it is reasonable to conclude that using passing path information to predict the final shooting outcome is both valid and practical. This implies that the outcome of a football shot is significantly influenced by the spatio-temporal path of the ball’s movement. Furthermore, the three models consistently yielded similar prediction results, further supporting the argument that passing path can indeed predict the shooting outcome in football games.

**Table 3:** Prediction results of the machine learning models.

Model	Logistic Regression (selected features)	Logistic Regression (all features)	Random Forest (all features)	ANN (all features)
Accuracy	69.3%	69.4%	69.0%	69.5%
Specificity	68.1%	68.1%	68.8%	68.1%
Sensitivity	73.0%	73.1%	69.2%	73.5%
AUC	76.0%	76.1%	75.6%	76.6%

The results of the Logistic Regression model using both sets of features (i.e., 18 features and 13 features) exhibit similarities, as presented in Table 3. Since no significant changes were observed between the results obtained from the two feature sets, this work will focus on the results of Logistic Regression using all the features. The main output of the Logistic Regression model (using all the features) is provided in Table 4, which includes detailed estimations of the features. Statistically significant features at the 0.01 confidence level include the *ratio of passing length*, *start distance*, *shot distance*, *y range*, *moving directness*, *passing ratio*, *shot index*, and *acceleration index*.

**Table 4:** Results of Logistic Regression.

Features	Estimate	Std. error	Odds ratio	Z-value	P-value
(Intercept)**	0.855	0.148	2.350	5.791	0.000
*Overall path length*	− 0.049	0.288	0.952	0.171	0.864
Ratio of passing length**	− 0.447	0.127	0.640	− 3.507	0.000
Start distance**	0.627	0.167	1.873	3.768	0.000
Shot distance**	− 8.780	0.327	0.000	− 26.858	0.000
*Average distance*	0.237	0.238	1.268	0.999	0.317
*x range*	0.153	0.169	1.165	0.905	0.365
y range**	− 0.841	0.129	0.431	− 6.531	0.000
Moving directness**	− 1.341	0.332	0.262	− 4.039	0.000
Possession time*	− 0.720	0.340	0.487	− 2.115	0.034
*Overall moving speed*	− 0.272	0.330	0.762	− 0.823	0.411
*Direct speed*	− 0.374	0.355	0.688	− 1.053	0.292
Passing ratio**	1.325	0.289	3.760	4.579	0.000
*Number of players*	0.343	0.222	1.410	1.545	1.222
*Centralization of possession time*	0.157	0.099	1.169	1.589	0.112
Centralization of passes*	0.186	0.089	1.204	2.095	0.036
Shot index**	1.314	0.107	3.721	12.317	0.000
Acceleration index**	− 1.316	0.354	0.268	− 3.721	0.000
*Attack intensity*	− 0.051	0.278	0.950	− 0.185	0.853

Features with ** and * are statistically significant at the level of *p*  < 0.01 and *p*  < 0.05, respectively.

Unfortunately, the Logistic Regression model cannot directly provide the ranking of feature importance, as mentioned in the last paragraph of “Methods” section. Although one might argue that the magnitude of the feature coefficients can serve as an indicator of feature importance as mentioned in the last paragraph of “Methods” section of this paper, it is still not robust enough as it does not consider the variation of each feature or the influence of the order in which features are entered into a stepwise model. Therefore, this paper did not provide a ranking of feature importance for the Logistic Regression model.

**Figure 4:**
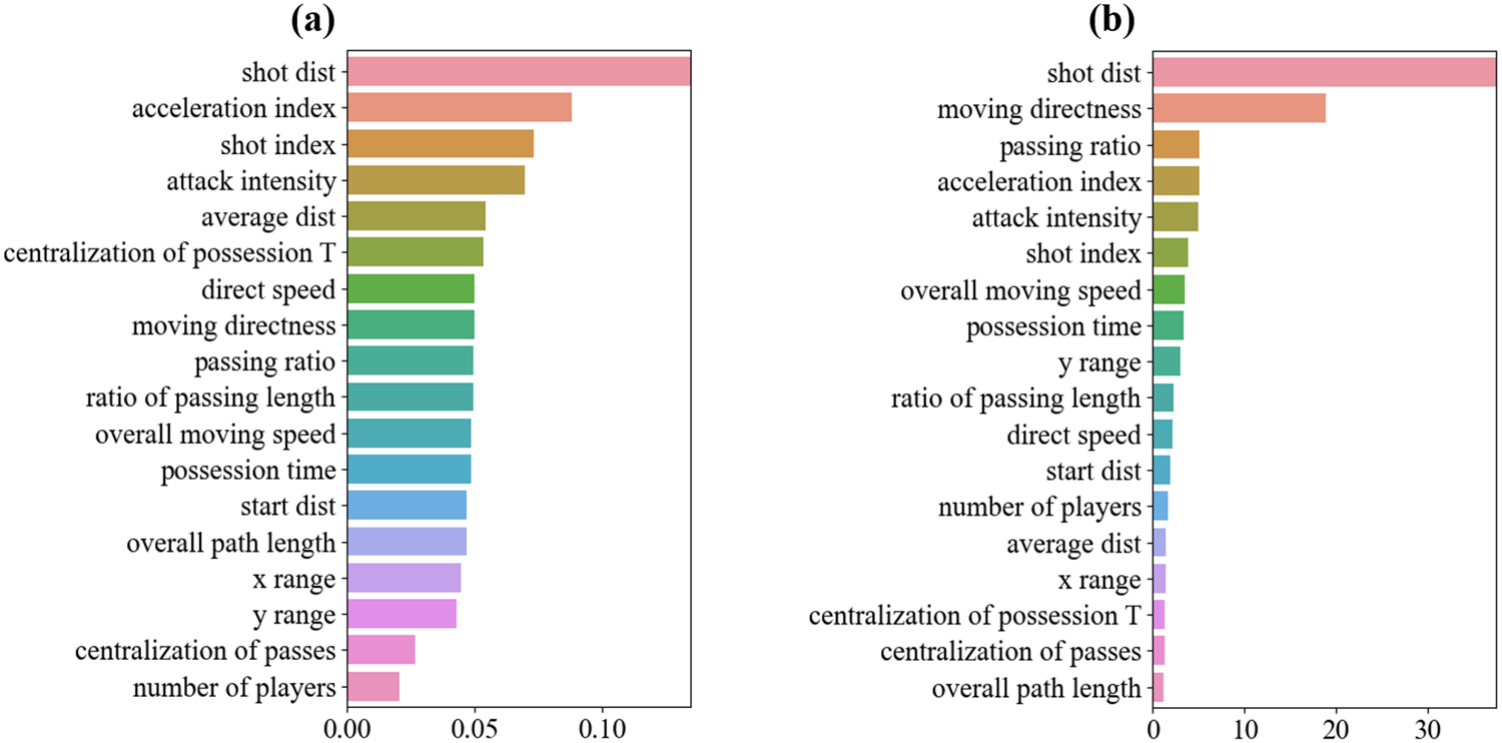
Ranking of feature importance. (**a**) Feature importance ranking based on Random Forest model (**b**) Feature importance ranking based on ANN model.

To obtain the ranking of feature importance, it is possible to turn to the models of Random Forest and ANN, as illustrated in Fig. 4. The top five important features in the Random Forest model are the *shot distance*, *acceleration index*, *shot index*, *attacking intensity*, and *average distance*. On the other hand, the top five important features given by the ANN model are the *shot distance*, *moving directness*, *passing ratio*, *acceleration index*, and *attacking intensity*. By summarizing the results obtained from all three models, it becomes evident that positional information related to the occurrence of shooting (e.g., *shot distance*, *shot index*, *acceleration index*) is the dominant factor in predicting the outcome of shooting in football games. This conclusion aligns with common knowledge in football, where a closer shooting position to the target is more likely to result in a goal. It also corresponds to previous studies on shooting positions^15,25,28,31,33^. However, it is important to note that the shooting position may also be influenced by the preceding ball movement path from one position to another. Simply focusing on letting the right person shoot the ball from a good position does not encompass the entire story of a football game. It is necessary to rerun the prediction models without considering the positional information of shots. By comparing the new prediction results with the results presented in Tables 3 and 4, as well as Fig. 4, a deeper understanding of how the passing path determines shooting outcomes can be gained.

### Enhanced prediction results

In the updated predictions, three features directly related to shooting position—*shot distance*, *shot index*, and *acceleration index*—were removed. Additionally, all other features that incorporated positional information of shooting were modified by excluding the last location of the passing path, which is the shooting position. The three models were then rerun using the updated feature set, and the prediction results are provided in Table 5. Although the accuracy, specificity, and sensitivity of all three models decreased by approximately 10% compared to the results in Table 3, they still showed relatively high values, close to or over 60%, for these metrics. The AUC index, which indicates the impact of passing path on the shooting outcome, remained above 60% even without information about where the shot occurred. Notably, the Random Forest model outperformed the other two models across all evaluation metrics, and its results are even comparable to those obtained when including shooting location information, as shown in Table 3.

**Table 5:** Prediction results of statical models (without information of shooting position).

Model	Logistic Regression (all features)	Random Forest (all features)	Backpropagation feedforward ANN (all features)
Accuracy	59.5%	65.2%	59.6%
Specificity	59.7%	63.8%	60.1%
Sensitivity	58.5%	70.2%	57.6%
AUC	63.4%	70.1%	63.7%

These findings suggest that, in addition to the final shooting position, features of the passing path—where the ball originates, when it arrives before the shot, and how it reaches the shooting position—significantly influence the outcome of the shot. A comparison of the results presented in Tables 3 and 5 also indicates a strong correlation between the final shooting position and the preceding ball’s movement from one location to another, thereby validating the hypothesis of this study: the passing path does predict the shooting outcome in football matches. These findings provide further perspective for the research domain of the ‘expected goals model’^14,15^. Extending the focus solely on factors such as the final shooting positions, shooting angles, and shooter’s/goalkeeper’s skills and performance, to incorporating the passing path in their analysis may significantly benefit this topic. Because the critical factor, shooting position, in the ‘expected goals model,’ is highly dependent on the preceding passing path. Furthermore, these findings provide scientific evidence for the endeavor of crafting offensive and defensive strategies by team coaches and specialists, who aim to identify the most effective passing paths conducive to successful shooting endeavors.

**Figure 5:**
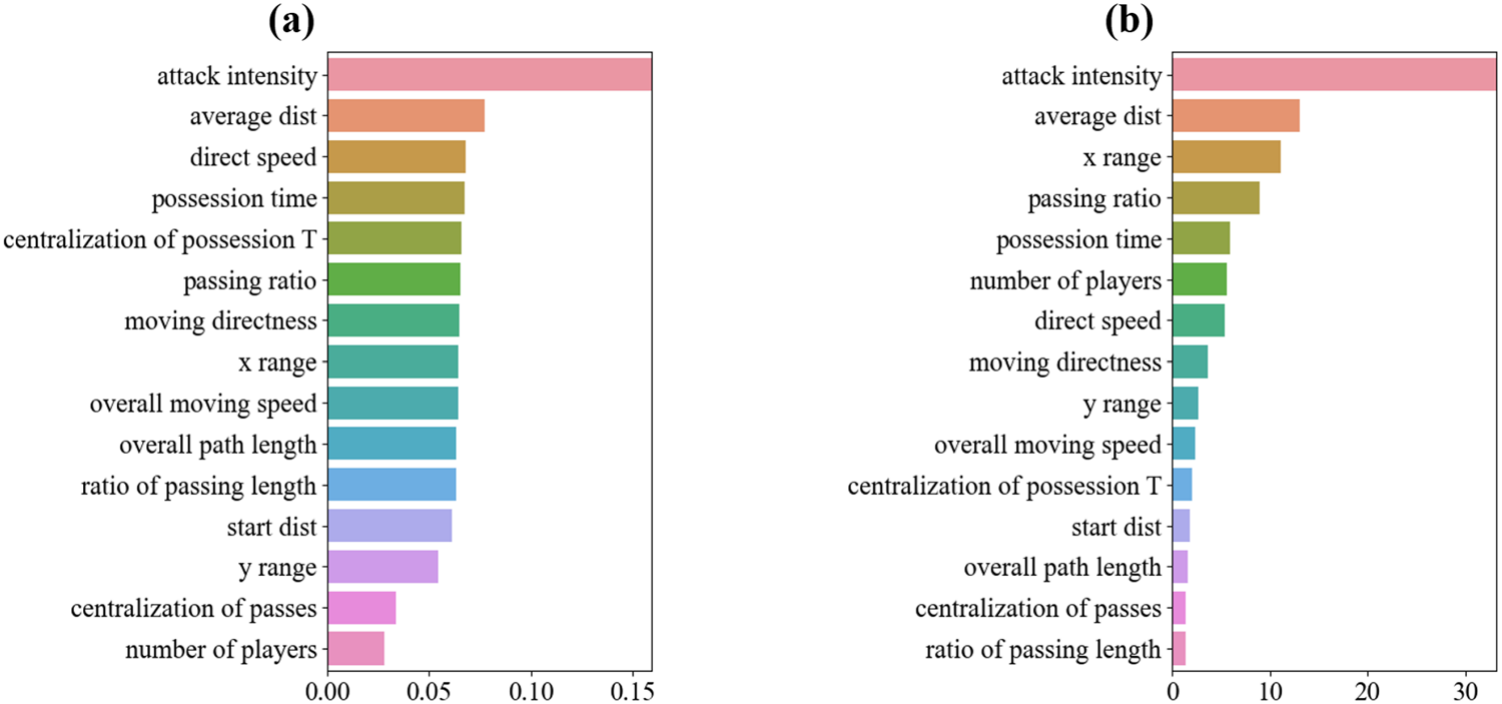
Ranking of feature importance (without the information of shooting position). (**a**) Feature importance ranking based on Random Forest model (**b**) Feature importance ranking based on ANN model.

As shown in Table 6, the results of the Logistic Regression model obtained by using the updated feature set indicated that the features of *average distance*, *x range*, *y range*, *possession time*, *passing ratio*, *number of players*, and *attack intensity* were statistically significant features at the 0.01 confidence level. The ranking of the feature importance of the models of Random Forest and ANN is given in Fig. 5. By summarization of the two sets of results in Fig. 5, the features of *attack intensity* and *average distance*, are the two most influential determinants in both results, which were also suggested as significant features with relatively higher coefficients in the outcome of Logistic Regression model as shown in Table 6. The feature of *attack intensity*’s significance suggests that attacking teams should take quick actions to move the ball to critical areas with high *shot indexes*. If possible, they should also aim to frequently move the ball across those critical areas and find an appropriate opportunity to conduct the final shooting action. By doing this, the team will significantly improve the probability of a successful shot. The feature of *average distance*, extended from previous studies, indicates that the closer a passing path (i.e., multiple positions without considering the shooting position) is to the attacking target, the more likely the shooting action will result in a scored shot.

**Table 6:** Results of Logistic Regression without information of shooting position.

Features	Estimate	Z-value	P-value
*(Intercept)*	− 0.129	− 1.201	0.230
*Overall path length*	0.131	0.448	0.654
*Ratio of passing length*	0.024	0.203	00.839
*Start distance*	− 0.101	− 0.566	0.571
Average distance**	− 3.193	− 13.816	0.000
x range**	2.294	13.886	0.000
y range**	− 0.528	− 5.008	0.000
*Moving directness*	− 0.091	− 0.227	0.820
Possession time**	− 1.260	− 4.157	0.000
*Overall moving speed*	− 0.194	− 0.444	0.657
Direct speed*	0.899	2.664	0.008
Passing ratio**	1.713	5.734	0.000
Number of players**	1.040	5.620	0.000
Centralization of possession time*	0.296	2.837	0.005
*Centralization of passes*	0.077	0.868	0.385
Attack intensity**	2.073	7.877	0.000

Features with ** and * are statistically significant at the level of *p*  < 0.01 and *p*  < 0.05, respectively.

In addition to the two most important features, *x range* of a passing path serves as another significant feature in predicting the shooting outcomes, ranked third in importance in ANN analysis, a relatively important feature in Random Forest analysis, and the second-largest coefficient in Logistic Regression analysis. The importance of *x range* suggests that attacking teams should try to move the ball over a relatively large range between the two goal gates (i.e., the *x* direction as shown in Fig. 3). This finding is surprising, especially considering a study reported by Buldu et al.^48^, who concluded that teams proficient at moving the ball parallel to the attacking goal (i.e., in the *y* direction as shown in Fig. 3) have greater potential for successful shooting. Clearly, this finding contradicts that notion to some extent. The feature of *passing ratio*, also called network intensity^15,23^, is another factor that can significantly affect shooting outcomes. This suggests that a series of quick and smooth passing actions (e.g., without being interrupted by duels) during ball possession is another important factor for successful shots. *Possession time*, serving as a crucial metric for evaluating a team’s performance and style of play^29,30^, is also proven to be significant in this study, but in a different way. While the overall possession time during a football match is commonly considered to be positively related to team performance, the possession time for each attacking attempt (i.e., the duration of each passing path) in this work is proven to be negatively related to shooting outcomes. This means that possessing the ball for a long duration before shooting is not advantageous for shooting effectiveness.

On the contrary, the *centralization of passes* regarding the level of inequality in the distribution of passing actions among different players is found to have little impact on shooting outcomes. However, this finding is not aligned with some empirical studies on passing network analysis which concluded that a team’s overall centralization of passing actions is highly linked to team performance^12,35,36^. The feature of *ratio of passing length*, measuring the proportion of passing distance within the *overall path length*, is also considered an unimportant feature in this analysis. This potentially suggests that the ball’s movement, either conducted by passing actions or other actions (e.g., dribbling), does not affect shooting outcomes. Additionally, the *overall path length*, which was concluded to be positively correlated with shooting outcomes^27^, is not a significant feature in this analysis. Furthermore, the *overall moving speed*, identified as strongly correlated with a football team’s attacking style and performance^32^, is found not to be a significant feature in determining shooting outcomes in this study.

Considering the prediction performance of the three models, one may argue that the results of Random Forest should attract more attention as it gave the best prediction results as shown in Table 5. Which might indicate it is more successful to capture or extract the most informative features in the passing path for shooting outcome prediction. This is also a good way to look at the results. If only consider the results of Random Forest model, the most important two features are *attack intensity* and *average distance*, while the *number of players*, *centralization of passing actions*, and *y range* were suggested as the three most unimportant features in determining the shooting outcome. All the other features were similar regarding the importance.

### Limitations of proposed method

While the proposed method yielded a satisfactory amount of knowledge, it is crucial to recognize several inherent limitations within it. Firstly, the features extracted from the passing paths are limited, and while the features used in this study have demonstrated their predictive capabilities for shooting outcomes, there may be other significant features that were not considered. Secondly, the model selection process was heuristic, and the tuning of the models may not have been optimal. Thirdly, this study did not account for the diverse styles and strategies of play observed in various leagues, as explored in prior research^49–51^. Consequently, it may not comprehensively capture the nuanced intricacies inherent in different playing styles across leagues. Finally, apart from the passing path, there may be many other factors that can potentially affect shooting outcomes, such as defenders’ positions, collective formations and movement, current game situations, and attack styles (e.g., corner kicks, counterattacks, free kicks), which were not considered in this study. Further comprehensive investigations of these variations are recommended for future research.

## Conclusions

This study investigates the impact of passing paths on shooting outcomes in football games. Eighteen features were extracted from the passing paths, providing insights into various aspects such as space, time, and collective behaviors regarding the ball passing and interactions among players. Three machine learning models were employed to establish the relationship between path features and shooting outcomes, enabling the modeling and prediction of shooting results. Prediction results suggested that the passing path can significantly affect the shooting outcomes in football games. Although the models yielded slightly different results regarding feature importance, consistent conclusions emerged, with shooting position being the most influential factor in shooting outcomes. Surprisingly, certain factors considered critical to team performance, such as *centralization of passing actions*^23,35,36^ and *overall path length*^27^, were found to be less important to shooting outcomes. When excluding the shooting positional information from the models, updated results suggested that the *attack intensity* and the *average distance* of the passing path were suggested as the most influential features that determine the outcome of shooting. Which means teams taking fast actions to move the ball in the areas of high probability of scoring a goal in a quick way can significantly increase the chance of successful shot. These findings highlight the significant effects of passing path features on shooting results, offering valuable insights into the key passing path features that influence shooting success. The insights will aid coaches and sports analysts in identifying effective attacking strategies and enhancing team performance.

## Data Availability

The datasets utilized in the present study can be accessed via figshare.com (https://figshare.com/collections/Soccer_match_event_dataset/4415000/5). Additionally, for a comprehensive understanding of the data employed in this research, readers are encouraged to refer to the paper titled “A public dataset of spatio-temporal match events in soccer competitions” by Pappalardo et al.^7^, which provides detailed insights into the dataset.
